# Sexual Behavior and Condom Use among Seasonal Dalit Migrant Laborers to India from Far West, Nepal: A Qualitative Study

**DOI:** 10.1371/journal.pone.0074903

**Published:** 2013-09-05

**Authors:** Kiran Bam, Rajshree Thapa, Marielle Sophia Newman, Lokesh Prasad Bhatt, Shree Krishna Bhatta

**Affiliations:** 1 District AIDS Coordination Committee (DACC), District Health Office, Silgadhi, Doti, Nepal; 2 Association of Medical Doctors of Asia (AMDA) Nepal, Damak, Jhapa, Nepal,; 3 James P. Grant School of Public Health, BRAC University, Dhaka, Bangladesh,; 4 Association of Medical Doctors of Asia (AMDA) -HAMI Project, Kathmandu, Nepal,; 5 Far Western Regional Health Directorate, Rajpur, Doti, Nepal; Alberta Provincial Laboratory for Public Health/ University of Alberta, Canada

## Abstract

**Background:**

Around 41% of Human Immunodeficiency Virus (HIV) cases in Nepal occur in seasonal migrant laborers. Dalit migrant laborers represent the largest proportion of reported HIV cases in the Far Western Region (*Sudur Pashchimanchal*, or Far West), Nepal. The study’s objectives were to assess sexual behavior, condom use status and HIV risk perception among Dalit migrant laborers to India from Far West Region, Nepal.

**Methods:**

The study was conducted among Dalit male migrant laborers aged 15 years and above who had migrated for at least six months of the last two years to India. For the sampling the village development committees (VDCs) from Achham, Doti and Kanchanpur districts of Nepal were purposively selected. The data were collected in March and April 2011 via ten in-depth interviews and four focus group discussions and analyzed using content analysis.

**Results:**

Poor socio-economic status, caste-related discrimination, and lack of employment opportunities push large groups of young Dalits to migrate to India for employment, where they engage in sex with female sex workers (FSWs). The participants described unmarried status, peer influence, alcohol use, low-priced sex with FSWs and unwillingness to use condoms as common factors of their migration experience. Lack of awareness on HIV/AIDS was common among study participants. Awareness of HIV/AIDS and faithful, monogamous partnerships are reported as factors influencing safer sexual behavior.

**Conclusions:**

Dalits are an especially vulnerable population among migrant laborers and may be over-represented in new HIV infections in Nepal. Comprehensive surveying and health promotion programs targeted to this population are urgently needed and potent methods of stopping HIV spread.

## Introduction

Human Immunodeficiency Virus (HIV)/Accquired Immune Deficiency Syndrome (AIDS) is a global threat. According to the Joint United Nations Program on HIV/AIDS (UNAIDS), an estimated 33.4 million people worldwide are HIV-positive [1]. In Asia, 4.7 million people were living with HIV as of 2008, including 350,000 people infected that year [1].

After the first reported case of HIV in 1988 in Nepal [2], the epidemic has gradually increased. Numerous socioeconomic and political factors increase national vulnerability to HIV, including poverty, unemployment, illiteracy, poor education, armed conflicts, internal and cross-border migration, gender inequalities, injecting drug use, commercial sex work and sex trafficking, and stigma related to sex and sexuality [2]. National Center for AIDS and STD Control (NCASC) estimated HIV prevalence as 0.49% of the adult (15-49 years) population. As of December 2009, there were 15,043 documented cases of HIV and 2,729 cases of AIDS in Nepal [3]. In total, 70,000 people are estimated to be living with HIV/AIDS, of which about 80% are unaware of their status [4].

Nepal is one of the world’s least developed countries. The country experiences considerable seasonal labor-related migration [5], mostly to India [6]. An estimated 967,000 to 1,511,000 seasonal migrant laborers depart Nepal annually [7]. Migration involves 25% of the Nepalese adult male population [8]; per the Nepal Labor Force Survey (2008), 44% of households have at least one absentee living either abroad or within the country [9].

Migration is very high in the West, Midwest, and Far West Regions of Nepal. The Integrated Bio-Behavioral Survey (IBBS) shows the median age of migration is low [10]. One in four (25%) first migrate before reaching 18 years, and 50% migrate before age 22 years [10]. In the hill districts of Far West Region, almost 80% of adult males from 80–90% households migrate to India for manual labor work [11]. Typically, men migrate in the summer and come back in winter.

Migration assists the spread of any infectious disease [12]. The topography of Far West Hills, environmental degradation, poverty and economic migration are linked, and they combine with other factors to increase vulnerability to HIV [13,15]. Not only does migration facilitate the rapid spread of a virus along so-called *corridors of migration*, but also causes behaviors and situations that facilitate transmission [14]. Seasonal migrant laborers have been identified as a “bridge population,” who behave in ways that spread HIV in the general population. A 2010 study among Nepalese migrant laborers traveling to Indian cities (a common destination) found 32.8% (up from 27% in 2006 and 22% in 2008) men engaged in unprotected sex in India, often with sex workers [10]. As a result, migrant laborers account for almost 40% of all new infections, per the 2010 United Nations General Assembly Special Session (UNGASS) [13]. National data identifies the highest burden of people living with HIV(PLHIV) among estimated seasonal migrant laborers (41%), followed by injecting drug users (34.7%), female partners of HIV-positive men (21%) and clients of sex workers (16%) [5]. HIV prevalence of migrants is reported to be 1.4% (Mid West) and 0.8% (Far West). But the prevalence in migrants’ wives is 3.3% [10], i.e. roughly two to four times higher than in the population overall. This increased prevalence is consistent with evidence that as many as one in every ten male migrants returning from Mumbai is HIV-positive [1]. Laborers’ vulnerability to contracting Sexually Transmitted Infections (STI) and HIV affects spouses and children in turn [2,4]. Low-risk women (rural and urban) account for 26% of total new infections, per the 2010 UNGASS [13].

Moreover, adequate knowledge of HIV/AIDS among migrants appears to be the lowest in the nation: 15.8% in the Mid West and Far West Regions, and 17.2% in the Western Region [13]. This increases risk to spouses for infection through decreased opportunities for self-protection, particularly because condom use between migrant laborers and their spouses at home is inconsistent [10].

The Dalits, who are 13.05% of the population [16], may be especially vulnerable. They are isolated because of untouchability [17], Nepal’s ethnic and caste system practice of ostracism and segregation of a minority due to the perception that they contaminate members of other social groups. Among all Nepalese social groups, they have the highest proportion of men migrating from Nepal to India [18]. Dalits migrate inside and outside of the country in part to escape caste-based discrimination [19]. As a result, they are a highly mobile population, with 59.7% migrating to India. Overall, they account for 31% of the total migrants to India [18]. Among Dalits, 90% live below the poverty line and only 10.7% are literate [18]. These factors likely increase Dalits’ HIV risk. However, no study focusing on the migration and HIV risk among Dalit migrants has ever been conducted. This limits our understanding of their vulnerability.

The objectives of this study were to assess sexual behaviors, lifestyle choices, and perceptions of HIV/AIDS among Dalits who migrate to India from Far West Region, Nepal. The study aims to understand the connections between migration and HIV risk among a sub-population that differs in vulnerability from the overall Nepalese population or migrant population. Because no previous research on HIV/AIDS among Dalits has been done, a study that contextualizes their vulnerabilities and perceptions is crucial to creating appropriate HIV/AIDS education and interventions for them.

A qualitative study is particularly useful as a first step, as it asks “how” and “why” questions that elicit patterns of behavior from the affected population themselves [20]. As the study is inductive, not deductive, there is no hypothesis to test [20]. However, the result can be the basis of hypotheses which can be tested via epidemiological surveying or community health interventions. Additionally, complex public health issues that involve personal behavior change and gaps between public perception and scientific knowledge are often well-addressed by community-based, participatory research (CBPR) [19]. A qualitative study is useful baseline information for such a study.

In order to ensure the quality of this qualitative work, this publication was written in compliance with the Consolidated Criteria for Reporting Qualitative Research (COREQ) standard [21], which compiles best practices for qualitative studies.

## Methods

This study was a two-stage process. First, four focus group discussions (FGDs) of Dalit male migrant laborers were conducted. Later, the results of these discussions were the basis of questions for ten in-depth interviews.

### Study area and period of data collection

The study purposively sampled from villages in three village development committees (VDCs), from three districts of the Far West Region. Of these, two villages are hilly region: Achham (in *Saubhageswor* VDC) and Doti (in *Kadamandau* VDC). The other, Kanchanpur (in *Baani* VDC), is terai region. Kanchanpur was selected because it borders India, and Achham and Doti were selected because they are in the HIV epidemic zone of Nepal [4]. The study period was March and April 2011.

### Inclusion criteria

The study sampled participants who are likely to migrate, basing inclusion of reported median age of first migration. In Achham district (Far West Region), this age is about 16 years; in Doti district, around 19 years [10]. Dalit men aged 15 years and over who had migrated within the last two years for six months or more and who were residents in the study areas during March–April 2011 were eligible to enroll.

### Exclusion criteria

Due to the low number of female migrants, only male migrants were eligible.

### Ethical issues

This study received ethical approval for the study and its verbal informed consent process from scientific review committee of the Samata Foundation (a Dalit youth-led organization in Lalitpur, Nepal) and the District Health Office in each district, following the ethical norms of the Nepal Health Research Council (NHRC), Kathmandu, Nepal.

Written consent was not acquired to ensure participant anonymity and no personal identifiers were recorded. A verbal consent process describing the objectives of the study, the participant’s involvement, benefits, risks, and confidentiality were completed with each participant in the local language (*Doteli* or *Achhami*) for the participation in the research and recording in the audiotape. They were informed that their participation was voluntary and that they were free to refuse to answer any question or to withdraw at any time.

Interview data did not contain the names of participants and all data were anonymized before analysis. The audio tapes were destroyed after the transcribing the notes. Each process was documented by the person administering consent in the presence of a witness and endorsed by the witness. The participants were provided Nepalese Rupees (NRs) 100 (US$1.12) as transportation compensation to and from the study area.

### Research entrée

The lead researcher was working as a District AIDS Coordination Committee (DACC), coordinator in Doti district and belongs to the Far West Region. For this reason, he was familiar with the local culture and community. The lead researcher was male and spoke the local language fluently, and these factors helped with building rapport with male migrants. The researchers were linguistically fluent and culturally competent. At the time of the study, the research team had been conducting HIV/AIDS work in the Far West Region for three or more years.

The study relied on various organizations to act as starting points for recruitment and to host discussion meetings. The Far Western Regional Health Directorate and District Health Office of all districts assisted in coordination with these organizations, which also allowed for easier entrée into the study area.

### Sampling technique

To develop the list of potential participants available in the VDCs during the study period, the research team carried out coordination meetings with HIV/AIDS organizations, especially those that prioritized the migrant population. Similarly, the researchers talked with the local community members.

At the first stage (FGDs), a purposive sampling technique was employed to ensure the representation from a comparative number of hilly and terai Dalit migrants. For in-depth interviews, the sample was at random.

### Recruitment, enrollment and informed consent

Within each VDC, we consulted with the local health organizations (with the assistance of the District Health Office) to construct a list of potential participants. From this list, we selected men at random to approach. We asked these men to join the FGD. All who agreed completed an informed consent process prior to the FGDs. All FGD were conducted inside the health facility.

The in-depth interview participants were first visited at their household and asked if they were interested to participate in the study. The research team provided options to all prospective interview participants: they could answer questions at their home or at the health facility the next day. Of ten participants, three opted for home interviews. The other seven were interviewed in a private room at a health facility. Some in-depth interview participants were identified during FGDs. These individuals were included in the FGD, and then approached for in-depth interviews afterward.

### Non-participation

Two men who showed interest in the in-depth interview and opted to meet at the health facility did not show up for the interview. One of these men could be traced later on, and he reported that he terminated his participation due to busyness. The other participant had temporarily left the area for undetermined personal reasons. All ten participants interviewed, however, completed the interview without withdrawing.

In the case of FGDs, the target was eight participants per group. At least twelve persons were contacted for each group prior to the FGD to compensate for any loss of participants who failed to show up. During the first contact, most participants agreed to participate. Five men expressed uncertainty due to personal circumstances (being busy or travel difficulties), and three of the total contacted refused to participate. Among those who agreed (n= 40), 36 participants attended the FGD. Among these, only 32 (8 per group) were recruited, to the total permitted under the study protocol.

### Data Collection Tools and Techniques

The study used multiple qualitative data collection methods (in-depth interviews and FGDs). These techniques allow for the systematic collection of insights and in-depth information about migration and HIV risk perception among the study population.

Ten in-depth interviews were completed using a semi-structured, open-ended questionnaire. The four FGDs used a FGD guideline. FGD guideline and in-depth interview guideline were prepared in English, translated it into the local language, and culturally modified it as per the Nepalese setting. Local languages (*Doteli* and *Achhami*) were used to avoid potential misinterpretation of questions.

The research team completed pre-testing to validate both guidelines among Dalit migrants in Geta VDC (Kailali district, Far West Region). A single FGD and three in-depth interviews were carried out as pre-testing. Pretested participants were similar to the study participants in terms of migration pattern, cultural context and residential proximity to the study districts.

After pre-testing, some culturally sensitive words referring to socioeconomic status and sex practices were omitted. References to anal sex and oral sex were excluded after pre-testing because participants refused to make distinctions as to which type of sex they had. Similarly, the local term, *Dhaal*, was substituted for more formal terms for *condom*. Additionally, questions pertaining to homosexual behavior were also excluded, because validation-step participants did not report this practice. This lack of disclosure may be because the behaviors were not practiced, or because pre-test subjects did not wish to disclose them. This ambiguity could distort results, which made this omission necessary.

In-depth guidelines were revised after preliminary analysis of each interview to include new themes and issues and to accommodate individual perceptions and experiences. The FGD guideline required minor revisions after pre-testing. Data from the pre-testing phase have not been included into the analysis.

All interviews were conducted in a private place, which facilitated dialogue. The FGD guideline was intended to extract more general ideas on the sexual behavior and condom use and In-depth interview was intended to explore personal experiences and perceptions of the individual participants.

All in-depth interviews and FGDs lasted for about one and a half hours. For the FGD, a local moderator was hired. In FGDs, the research team made efforts to balance the numbers of younger (15-24 years) and adult population (above 24 years). The FGDs focused on places of migration, knowledge, common norms and values, perceptions, sexual behavior, sexual experiences, and access to and utilization of HIV/AIDS services. These were used to clarify issues that had been raised in the in depth interviews. They were provided with free condoms and information education & communication (IEC) materials from the local health facility.

During the interviews, contextual anecdotes were used to draw out introverted or resistant participants. However, this proved mostly unnecessary, as most participants were willing to share extensive personal information. To ensure accurate comprehension, the researchers paraphrased interviewees’ answers throughout the interview. Researchers also summarized the major findings at the end of each interview. Similar techniques were used in the FGDs. The findings were summarized with participants at the end of each FGD.

### Data management and analysis

FGDs were audio-recorded and were also described in notes and diagrams. The recordings were transcribed immediately after each session. The data coders analyzed the transcripts site by site, across sites, and by themes. Major thematic areas and minor thematic areas were identified and in collaboration, a theoretical framework was developed to analyze the data [22]. Verbatim quotes were used when necessary to highlight specific issues.

After the eighth of ten in-depth interviews, data saturation was reached, and very little new information was obtained. Therefore the interviews were stopped after ten interviews. Similarly, after the four FGDs, our research objectives were met and hence we concluded the FGDs.

The individual interviews were also audio-taped with the participants’ permission. Field notes were taken from all the interviews. Data were analyzed manually on the same day of the interview. The data were coded through a consensus process. The in-depth interview themes were developed prior to the study’s start, but most major and minor themes were later extracted from the study. Data were checked for theme repetition. Theories of community-based participatory research were useful to analyzing perceptions of participants in comparison to empirical knowledge of HIV/AIDS [19]. In addition, looking glass self concept emphasizing relative social power, hierarchy and self-efficacy were important to understanding vulnerabilities [23]. Finally, this study used an informal analytic framework informed by the researchers’ extensive knowledge of local context; while no researcher is Dalit their long-standing research formed an informal ethnographic basis for formulating questions for the study.

## Results

We conducted four FGDs at three places: Kanchanpur (in *Bani* VDC), Achham (*Saubhageswor* VDC) and Doti (*Kadamandau* VDC). Since *Bani* VDC is geographically larger, two FGDs were carried out there. FGDs with eight participants each (n=32) and ten in-depth interviews (n=10) were completed. Characteristics of the FGD are in Table 1. Characteristics of in-depth interview participants are in Table 2.

**Table 1:** Characteristics of focus group discussion participants.

**Focus Group Discussion**	**Average age (years)**	**Have attended school past the primary level** **n (%)**	**Location**
I	24.6	4 (50.0)	Terai
II	35.4	4 (50.0)	Terai
III	22.4	5 (62.5)	Hilly
IV	38.2	4 (50.0)	Hilly

**Table 2:** Characteristics of in depth interview participants.

**Characteristics**	**Sub characteristics**	**n (%)**
Age group (years)	15-20	3 (30.0)
	20-25	4 (40.0)
	25+	3 (30.0)
Educational status	Can read and write only	2 (20.0)
	Primary	3 (30.0)
	Lower secondary	2 (20.0)
	Secondary	2 (20.0)
	Higher secondary & above	1 (10.0)
Area of residence	Hilly	5 (50.0)
	Terai	5 (50.0)
Marital Status	Married	5 (50.0)
	Unmarried	5 (50.0)

### Magnitude of migration

Migration is very common among the Dalit families in Far West. We found that typically one, two, or more members of the family were migrating habitually to India for work, with particularly heavy migration among Dalit families. It was hard to find any young Dalit males who have never migrated to India for employment. We found that during FGD most of the Dalits said they migrate yearly after crop harvesting, and return during the rice planting season.

### Places of migration

Participants reported their major places of migration in India were Delhi, Madras, Mumbai, Uttar Pradesh, Bihar, Maharashtra, and Kolkata. Delhi was the most prominent destination and was considered one the reliable areas of employment for the Dalit migrants. Dalits reported migrating to India because it borders Nepal, has no visa requirements, and is cheaply accessed by highway and railway travel. Most respondents report they work as mine workers, gate keepers, garment workers, household workers, drivers and factory workers.

### Reasons for migration

Poverty was identified as the main driving force for the migration. Unemployment in Nepal, stigma and caste discrimination are other prominent reasons for migration. Relatedly, some study participants responded that inability to continue their studies pushed them to migrate. The social influence of friends and neighbors who are also migrating is a pull factor to India. Finally, wages available to laborers are typically higher in India than in Nepal, which is another pull factor.

“Here in Nepal, either you don’t get work or even if you get work the payment is very low, so we follow the tracks of other friends and neighbors and migrate to India.”

(*Age 18, Kanchanpur, FGD participant 3)*

“There is no water for agriculture, and the foods produced from the land are insufficient for the whole year. So it is the responsibility of the male members of the family to engage in income-generating work, and there is no option but going to India to work.”

(*Age 24, Doti, IDI 2*)

“All family members excluding me have been in India at some time so I visit there several times. I think that I also have to move from here due to the lack of opportunity. I have studied up to the tenth class, but I cannot find work for myself in Nepal. It is better to migrate to India and earn money.”

(*Age 18, Achham, FGD Participant 6)*

### Knowledge of HIV and STI

All the in-depth interview participants and FGD participants had heard of HIV and AIDS. However, only two of the ten in-depth interview participants had detailed knowledge on HIV and AIDS. Almost all mentioned HIV could be transmitted by sexual contact and blood transfusion. Most were unsure about transplacental and parenteral transmission of HIV. Moreover, many participants believed HIV could be transmitted by kissing, mosquito bites or social contact with PLHIV.

When asked if HIV is curable, most of them were unsure. The most frequent response was that it might be curable. However, all participants perceived that HIV was a fatal disease.

Most of the migrant laborers had listened to media messages on HIV. However, they admitted to never listening to those messages carefully and grasping few of the facts.

“HIV is transmitted if you have many sexual partners, which is all I know about HIV and AIDS.”

(*Age 39, Achham, FGD participant 1*)

“I have heard shaking hands does not transmit HIV, but I think it is possible to transmit by mosquito bite or kissing, or by being very close to PLHIV for a long time.”

(*Age 45, Kanchanpur, FGD Participant 8*)

“HIV is a disease that you get if you do not use a condom.”

(*Age 29, Doti, FGD Participant 4*)

### Sexual behaviors of migrants

Questions on sexual behavior included only the heterosexual acts. Questions pertaining to homosexual behaviors were not included in the study after the pre-testing of study tools. The distinction between oral, anal, and vaginal sex was not made because the behavior was not accurately reported.

Visiting brothels was very common among Dalit migrants. It was common to have sex with a FSW while in India. Almost all the Dalit migrants reported that they had visited FSWs, either in groups or singly. When asked, “Have you ever visited a FSW while in India?” only six participants (6/42, or 14.3%) claimed they had never done so. Some participants in one FGD argued that some migrants did have sex with FSWs in Mumbai, but refused to disclose their behavior.

“Once people migrate to India, they also have interest of experiencing the red light district of the India at least one time during their visit.”

(*Age 21, Achham, FGD Participant 4*)

“Usually the married men with their spouses back home go for the commercial sex workers there in India. I am younger and unmarried, but I sometimes visit the sex worker when I have sexual desire.”

(*IDI, Doti*)

“I had never had sex while I was in Nepal. When I went to India for work, my friends and relatives influenced me, and I visited there [the brothel] several times.”

(*Age 18, Kanchanpur, FGD participant 3*)

The monetary value of sex work in India was reported to range from NRs 50 (US$0.56) to NRs 10,000 (US$112.20) per night. Almost all participants reported visiting the cheaper places, ranging from NRs 50 (US$0. 56) to NRs 100 (US$1. 22) per night. Some of the migrants reported to have visited expensive sex workers, paying NRs 5000 (US$56.10) per night to have the experience of expensive services. However, the sex workers’ price starts from NRs. 50 (US$0.56).

“Money is never a big problem to have sex in India, as you can get number of call girls only just for rupees 50 per night, and if we don’t have money, then our friend can easily lend money for sex.”

(*IDI, Achham*)

“Older women charge you a lower price than that of the young girls.”

(*Age 26, Kanchanpur, FGD Participant 5*)

When asked about the frequency of visit to sex workers, FGD participants in Achham laughed and said it depends upon the person. There are some people who visit sex workers daily, but most visited weekly, mostly during the weekend. Some visited the sex workers only occasionally. Those visiting occasionally reported visiting the more expensive sex workers. Those visiting on a daily or weekly basis reported visiting the sex workers with lower rates.

“When I was in India, at the beginning I used to occasionally visit the sex workers. Gradually we formed groups of friends, and after that we used to visit frequently, sometimes three times a week.”

(*IDI, Achham*)

When asked about the number of sexual partners, most of them responded that they used to change the partners every time they visit. It was rare to repeat the same sex partner.

Of course, everyone wants to change the women every other day.”

(*Age 20, Kanchanpur, FGD Participant 2*)

### Sexual behavior and substance use

Alcohol is found to be the catalyst of migrants sexual behavior. Almost every participant had a habit of drinking alcohol prior to their migration to India. Alcohol intake was common among the Dalit community dwelling in the Far West Region(Alcohol drinking is culturally sanctioned among the Dalit community, and non-drinkers are rare in this community. Only two of the total participants reported to have developed the habit of drinking *after* first visiting India.) Men reported that alcohol increased their sexual desire and propensity to engage in sexual behavior. They drank in groups and then visited brothels together.

“While drunk, sexual desire is greater, and we visit female sex workers. In such conditions, we don’t even use condoms.”

(*FGD Participants*)

Though marijuana is common among the migrants in Far West Region, drugs other than marijuana are uncommon These migrants reported using only marijuana in Nepal. Other substance abuse was not found. Migrants reported fearing taking drugs in India because they perceive that laws are stricter in India than in Nepal. They also reported lacking access and unaware to illicit substances in India, as they cannot pay and lack of knowledge on such drug availability.

In Nepal, we sometimes take gaanja [marijuana], but in India that is not possible. It’s very tight[controlled] in India and we can’t take such risks in another country.

(*Age 28, Achham, FGD Participant 2*)

### Sexual behavior and condom use

Condom use was uncommon and inconsistent among the migrants in India. Almost all participants who reported having sex with FSWs in India reported no use or inconsistent use of condoms. This was due to alcohol consumption, which impaired decision-making. It was also due to their belief that condoms reduce sexual pleasure and that they were at low risk of contracting HIV. Most of the time they visited FSWs while drunk, and declined to use condoms due to their impaired perception of risk. Most were aware that condoms reduce the chances of HIV transmission, but used them only when they suspected a particular FSW to be HIV-infected. They believed that healthy and good-looking women were unlikely to be HIV-positive and therefore did not find it necessary to use condoms with these FSWs.

They said that the FSWs in India have become aware of condoms and that FSWs knew more than them about condoms. The more expensive FSWs pressured them to use condoms, but FSWs seeking cheaper rates did not raise the issue. Migrants did not want to use condoms because they strongly believed that condoms will reduce sexual pleasure. The statement “condoms reduce sexual pleasure” was repeated frequently in the in-depth interviews and FGDs.

“Using a condom during sex is just like putting the plastic over the tongue while eating. The real taste of sex cannot be experienced by using condoms.”

(*IDI, Achham*)

“While drunk, we do not carry condoms, and also we are not in the state to think about condom use. Even though the sex worker suggests using condoms, I don’t use it.”

(*IDI, Doti*)

While asking if it was feasible for them to carry condoms all the time, two FGD participants said that they always carry condoms with them and also showed the condoms. The other participants, mostly married men, responded that carrying condoms was not always feasible. These participants feared that possessing condoms would communicate something negative to relatives who happened to see them.

When asked about the process of condom use, most migrants (18/32) did not know the exact method for correct condom use. These were mostly younger participants (15-24 years). Some (5/32) respondents, mostly 24 years and older, were aware about the exact way to use condoms. Among those who answered correctly, most responded they learned the method from the health workers in the area.

Migrants who were married replied that, although they sometimes use condoms during sex with FSWs, they never used condoms during sex with their wives. They reported that they did not feel it was necessary, as their wives had never asked them to.

“I never use condom with my wife, first because I don’t think it’s necessary, as I am not carrying any disease; and second, if I use condoms, my wife will suspect me of infidelity.”

(*IDI, Doti*)

### HIV risk perception

When asked if they could possibly contract HIV, only a few of the participants said yes. Others perceived themselves to be healthy and they will not contract HIV, as they did not experience any symptoms of AIDS. They perceive themselves to be healthy and free from HIV/AIDS, and therefore they perceived themselves to be unlikely to contract the virus in the future, despite risky behavior. Though some reported to have experienced symptoms of STI, they did not perceive themselves to be vulnerable to HIV and AIDS. They said having an STI is common, and they perceived that STIs heal soon, mostly without any medicine.

### Voluntary Counseling and Testing (VCT) services

When asked if they have ever heard of VCT and if they could state the services available through VCT centers, only three participants from Kanchanpur and five participants from the Achham had factually accurate knowledge about the purpose and availability of VCT. Others haven’t even heard of VCT. Those who knew about VCT shared with the other FGD participants that it is a process of knowing one’s HIV status. However, only three in-depth interview participants had undergone VCT. Of these three, two revealed they had tested positive for HIV. Others who knew about VCT but never utilized the service said that they did not use the VCT because they did not think that they are at risk.

After the purpose of the VCT center was described in brief, the participants were asked if they would ever go for VCT. The young migrants (15-24 years) showed their interest to utilize the service. They also demanded such services should be more easily accessible to the migrant population in particular. However, participants aged 40 years and above responded that they were not having any serious sexual problems to date, and therefore did not think they require such services.

“VCT services should be made available at the entry and exit point of India and Nepal. Moreover mass awareness focusing safe sexual behavior should be promoted to reduce the risk of HIV.”

(*Age 18, Kanchanpur, FGD* participant *3*)

### Programs on HIV/AIDS for migrants

The participants agreed that programs for the prevention of HIV/AIDS are increasing in the region; however they felt there is not any such program that can effectively address the need of the young migrants at the border areas. Participants reported dissatisfaction with HIV/AIDS service providers. They said programs have yet to reach the target population. Some young migrants did not know any sites for HIV/AIDS services in their area, which limits their access.

“In Far West Region, there are very few preventive programs for HIV/AIDS, and we Dalit migrants are not getting those services.”

(*Age 29, Kanchanpur, FGD participant 7*)

Most participants stressed that addressing the needs of young males who migrate to India in the future. Availability of condoms, counseling of migrants or potential migrants, and accessible VCT services at border areas were identified as migrants’ needs.

They also shared that awareness programs on HIV/AIDS are more common in India than in Nepal. Indian programs include information on HIV/AIDS through text messages to mobile phones, billboards at the major areas of the city, programs on safer sex awareness, and advertisement through television and radio. These could be replicated in Nepal.

One of the FGD respondents said that the programs are limited to just poster and pamphlet distribution, and that was not enough. Such programs should focus on behavior change through availability of services, such as provision of adequate condoms and counseling services.

“There are programs related to awareness but they only distribute pamphlets, posters and books, which did not help them in real experience. Once, when I asked for the condoms from such HIV/AIDS project in the region, they did not give me condoms.”

(*Age 26, Kanchanpur, FGD Participant 5*)

### Stigma and discrimination associated with HIV/AIDS

Stigma and discrimination against PLHIV is still common in the Far West Region. PLHIV are not provided proper care and support. Rather, their families and communities isolate them, fearing transmission of HIV through social interactions with HIV-positive people, and force them to live in unhygienic places.

This stigma and discrimination are deep-rooted in the Dalit community. It can be associated with the poor knowledge of the routes of HIV transmission. Most participants still believed HIV is transmitted through interaction with the PLHIVs. Some believed the disease could be transmitted just by touching a PLHIV.

“My father and mother both died of HIV/AIDS. When we knew about their status we did not let them stay with us. We made a small room at Goth [where cows and buffalos are kept] and let them stay there. We did not allow them to come outside, because we feared that the disease would be transmitted to us and our children.”

(*IDI, Kanchanpur*)

“My brother died of AIDS two years back. When he died, all of the people were afraid and no one even touched his body, because of the fear that they will get the disease through germs from his body.”

(*IDI, Achham*)

## Discussion

A key focus of this study was to seek knowledge of Dalit migrant laborers as a special subpopulation of Nepal. We found evidence of contrasts between Dalit migrant laborers, all migrant laborers, and the general population of Nepal. For example, a 2010 study among Nepalese migrant laborers to India found 32.8% of men engaged in unprotected sex with sex workers [10]. However, our study found that at least 85% of Dalit migrants had sex with FSWs in India (This may under-represent the true number, as some FGD participants alleged other participants did not accurately disclose their behavior). The large majority of these reported not using condoms. In other words, Dalit men reported engaging in high-risk behavior with FSWs much more than other migrants. This suggests that a particularly high rate of HIV infection may exist among Dalits.

Anecdotally, two of the three men who reported being tested said they were HIV-positive. This supports the idea that HIV prevalence among Dalit migrant laborers may be much higher than among migrants overall, which is reported to be 1.4% (West Region) and 0.8% (Far West Region) [10]. Migrant laborers suffer four in ten of every new HIV infections in Nepal [13]; Dalits, who are 13.05% of the total population [16], may be over-represented among new infections. Notably, the exceptionally low rate of VCT among the study participants (3/32, or 9.4%) suggests they are less likely to be diagnosed than 20% of all HIV-positive people who are aware of their status nationwide [5].

Dalit migrant laborers also reported low knowledge of HIV/AIDS. Knowledge of HIV/AIDS among migrants is 15.8% in the Midwest and Far West regions, and 17.2% in the Western region [10]. Our study found that five of 32 participants, or 15.6%, were aware of how to use a condom, less than ten percent were aware of VCT, and very few understood they were vulnerable to contracting HIV. These responses suggest knowledge among Dalits is lower than among the general population in Nepal. Notably, many migrants said they want HIV/AIDS services, including education and VCT, despite stigma against HIV-positive people.

The qualitative methodology allowed the opportunity to explore the relationship between migrant laborers’ use of sex workers in India to substance use and to finances. We found that poverty in Nepal drove migration behavior and that there is a dynamic relationship between male friendships, alcohol consumption, low-priced sex with FSWs, and HIV transmission among migrant laborers in India. This new finding is subject to further quantitative investigation using social network theory [24,25].

We also found that migrant laborers typically establish drinking habits prior to migration to India (which suggests an early age of first alcohol use, as the mean average of first migration is 19 years or less). We also found that alcohol use drives visits to sex workers and unsafe sex practices. However, we found that use of other illicit substances was unrelated to the sexual practices of study participants, as they rarely used these substances in India for legal and financial reasons.

The study is reported using the COREQ standard for qualitative research [21]. While metanalysis of qualitative studies is difficult, we aim to inform future surveying and community-based research through a high-quality reporting of data.

### Limitations

This study is based on self-report of participants. This is subject to recall bias. To minimize bias, questions were asked more than once, answers were repeated back to participants, and findings were reviewed at the conclusion of interviews and FGDs. For cultural reasons, participants were unwilling to discuss anal, oral, and all non-heterosexual sex, which was a limitation in self-reporting.

The study was also limited by “faking good,” a phenomenon where participants disguise or downplay behaviors associated with social stigma. Notably, some participants denied sexual activity with FSW, and this was refuted by their fellow FGD participants. This signal “faking good” took place in the studio (although in this case unsuccessfully), and this may indicate that responses to other questions were also subject to intentionally sanitized answers.

Asking participants review the data after transcription and analysis were infeasible due to limited resources and participant mobility. This may reduce the study’s face validity. The study’s other major limitation was that it does not crosscheck the statements of male migrant laborers with their wives, nor with the FSWs they visited. This was omitted due to the logistic challenges and ethical issues such a process imposes.

## Conclusions

This study’s findings are consistent with other published research on “bridge populations” in infectious disease epidemics. Studies by Morris, et al. [26] and Entz, et al. [27] have proposed migrants and other mobile individuals are bridge populations who spread HIV infection from high-risk to low-risk populations. High-risk sexual behavior in India [28–30], coupled with inconsistent use of condom inside and outside of marital relationships, increases husbands’ and then wives’ vulnerability to HIV.

Migrants appear to be a core group involved in high-risk sexual behaviors in the Far West Region of Nepal. The hypothesis of high rates of HIV infection in Dalits, arising from our study, is subject to empirical testing. Specialized attention to Dalits is both urgently needed and a probable potent method of stopping epidemic spread. A comprehensive study that documents the castes of people with new HIV infections would provide valuable information for targeting Nepalese subpopulations through cost-effective, culturally competent target programs.

Discouraging migration via improved economic empowerment in Far West Region may have a meaningful effect on HIV infection rates. Encouraging savings or remittance of wages in place of purchasing sex; special harm reduction-based strategies that encourage interest in high-priced, less-frequent, sober and safer sex with FSWs; or new standards of healthy drinking behavior and safer sex among male peer groups might be meaningful ways to reduce HIV transmission. Exploration via community-based participatory research or health promotion research is feasible. Additionally, this is a basis for well-informed quantitative surveying to further describe the relationship between these elements.

HIV/AIDS awareness campaigns should target youths who are marginalized or vulnerable due to poverty, migration, and other factors. Prevention programs should focus on high-risk behavior associated with commercial sex work and on high-risk behavior within established sexual partnerships, with a focus on consistent condom use with all partners, including wives. Our study suggests that coupling information on HIV/AIDS with information on alcohol use may be beneficial, but that messages on other substances may be irrelevant to the majority. This could inform and corroborate a comprehensive survey-based assessment of substance use and sexual activity among Dalits. Integration of messages on alcohol and sex into clinical and educational practices also is likely to be useful.

Smith-Estelle and Gruskin [29] reported migration, health status, gender-based discrimination, and access to education have an impact on HIV vulnerability among rural women from migrant communities in Nepal. A study by population council and United Nations Development Program (UNDP) showed a significant association between spousal migration and women’s HIV status [28]. A CARE study found that in one high-migration district, nearly 50% of suspected PLHIVs (34 out of 71 cases) were migrants [30]. It was also reported that the wives of the migrants do not consider them to be vulnerable to HIV as they do not believe that their husband are having sex with other partners [31]. Our study did not include information on wives’ perceptions or behavior but confirmed high-risk male behavior that can endanger their female partners. Further studies on the Dalit migrant laborers’ female partners are an important component of comprehensive programming.

Finally, we found that Dalit migrants want information on HIV/AIDS despite stigma against affected persons. Availability of VCT services should be expanded and intensified. Outreach should include comprehensive programs to high-risk populations.
